# Post-composing ontology terms for efficient phenotyping in plant breeding

**DOI:** 10.1093/database/baaf020

**Published:** 2025-03-21

**Authors:** Naama Menda, Bryan J Ellerbrock, Christiano C Simoes, Srikanth Kumar Karaikal, Christine Nyaga, Mirella Flores-Gonzalez, Isaak Y Tecle, David Lyon, Afolabi Agbona, Paterne A Agre, Prasad Peteti, Violet Akech, Amos Asiimwe, Eglantine Fauvelle, Karima Meghar, Thierry Tran, Dominique Dufour, Laurel Cooper, Marie-Angélique Laporte, Elizabeth Arnaud, Lukas A Mueller

**Affiliations:** Boyce Thompson Institute, 533 Tower Rd, Ithaca, NY 14853 , USA; Boyce Thompson Institute, 533 Tower Rd, Ithaca, NY 14853 , USA; Plant and Environmental Sciences, Pee Dee Research and Education Center, Clemson University, 2200 Pocket Road, Florence, Clemson, SC 29506, USA; Boyce Thompson Institute, 533 Tower Rd, Ithaca, NY 14853 , USA; Boyce Thompson Institute, 533 Tower Rd, Ithaca, NY 14853 , USA; School of Integrative Plant Sciences, Section of Plant Breeding and Genetics Cornell University, 236 Tower Road, Ithaca, NY 14853, USA; Boyce Thompson Institute, 533 Tower Rd, Ithaca, NY 14853 , USA; School of Integrative Plant Sciences, Section of Plant Breeding and Genetics Cornell University, 236 Tower Road, Ithaca, NY 14853, USA; Boyce Thompson Institute, 533 Tower Rd, Ithaca, NY 14853 , USA; Boyce Thompson Institute, 533 Tower Rd, Ithaca, NY 14853 , USA; Boyce Thompson Institute, 533 Tower Rd, Ithaca, NY 14853 , USA; Division of Environmental Genomics and Systems Biology, Lawrence Berkeley National Laboratory, 1 Cyclotron Road, Berkeley, CA 94720, USA; Texas A&M Agrilife Research Center, 2415 Business Hwy 83 E, Weslaco, TX 78596, USA; International Institute of Tropical Agriculture (IITA), PMB 5320, Oyo Road, Ibadan, Oyo State 200001, Nigeria; International Institute of Tropical Agriculture (IITA), PMB 5320, Oyo Road, Ibadan, Oyo State 200001, Nigeria; International Institute of Tropical Agriculture (IITA), 8JW4+3Q6, Naguru E Rd, Kampala, Uganda; National Agricultural Research Laboratories, P.O.Box 7065, Kawanda, Uganda; CIRAD - PERSYST, UMR QualiSud, 73 rue Jean-François Breton, Montpellier F-34398, France; QualiSud, Université de Montpellier, Avignon Université, CIRAD, Institut Agro, IRD, Université de La Réunion, Montpellier, France; CIRAD - PERSYST, UMR QualiSud, 73 rue Jean-François Breton, Montpellier F-34398, France; QualiSud, Université de Montpellier, Avignon Université, CIRAD, Institut Agro, IRD, Université de La Réunion, Montpellier, France; CIRAD - PERSYST, UMR QualiSud, 73 rue Jean-François Breton, Montpellier F-34398, France; QualiSud, Université de Montpellier, Avignon Université, CIRAD, Institut Agro, IRD, Université de La Réunion, Montpellier, France; International Center for Tropical Agriculture (CIAT), Km 17, Recta Cali-Palmira, Colombia; CIRAD - PERSYST, UMR QualiSud, 73 rue Jean-François Breton, Montpellier F-34398, France; QualiSud, Université de Montpellier, Avignon Université, CIRAD, Institut Agro, IRD, Université de La Réunion, Montpellier, France; Department of Botany and Plant Pathology, Oregon State University, 2503 Cordley Hall, 2701 SW Campus Way, Corvallis, OR 97331, USA; Bioversity International, Digital Inclusion Lever, Via di San Domenico 1, Roma 00153, Italy; Bioversity International, Digital Inclusion Lever, Via di San Domenico 1, Roma 00153, Italy; Boyce Thompson Institute, 533 Tower Rd, Ithaca, NY 14853 , USA

## Abstract

Ontologies are widely used in databases to standardize data, improving data quality, integration, and ease of comparison. Within ontologies tailored to diverse use cases, post-composing user-defined terms reconciles the demands for standardization on the one hand and flexibility on the other. In many instances of Breedbase, a digital ecosystem for plant breeding designed for genomic selection, the goal is to capture phenotypic data using highly curated and rigorous crop ontologies, while adapting to the specific requirements of plant breeders to record data quickly and efficiently. For example, post-composing enables users to tailor ontology terms to suit specific and granular use cases such as repeated measurements on different plant parts and special sample preparation techniques. To achieve this, we have implemented a post-composing tool based on orthogonal ontologies providing users with the ability to introduce additional levels of phenotyping granularity tailored to unique experimental designs. Post-composed terms are designed to be reused by all breeding programs within a Breedbase instance but are not exported to the crop reference ontologies. Breedbase users can post-compose terms across various categories, such as plant anatomy, treatments, temporal events, and breeding cycles, and, as a result, generate highly specific terms for more accurate phenotyping.

## Introduction

Ontologies consist of controlled vocabulary terms, organized into directed acyclic graphs, with defined relationships between terms, such as “is_a” or “part_of” [1]. The terms expand from more general to specific concepts—from the root to the leaves of an ontology. To be most useful, ontologies must be relatively stable, yet allow flexibility to accommodate changing user needs. Traditionally, crop database curators have served a significant role in the development cycle of an ontology by reviewing, editing, and modifying terms while maintaining the integrity of the ontology and providing users with timely updates. Online forms can provide a simple way for the users to suggest new terms for addition. These suggestions are then reviewed by the expert database curators.

In this work, we focus on trait ontologies for plant breeding, as used, for example, in the instances of Breedbase [2]. Breedbase databases establish a digital ecosystem for plant breeding programs, streamlining data management and ensuring accurate information for making breeding decisions. Several breeding programs in Africa and Consultative Group on International Agricultural Research (CGIAR) centers [3], for example, have successfully used Breedbase for over a decade to manage their complex multidisciplinary datasets [4]. For greater flexibility, breedbase also works seamlessly with smartphone apps, such as PhenoApps [5], for real-time data acquisition and processing.

The Sol Genomics Network (SGN [6]), as the original database version of Breedbase, has pioneered the use of phenotype ontologies with the Solanaceae Phenotype Ontology [7], as a tool for categorizing and storing Solanaceae phenotyping data. For other crops, beginning with cassava (https://cassavabase.org/), Breedbase started working with the Crop Ontology (CO) project (https://cropontology.org/) [3]. CO hosts and develops ontologies for many crops that are bred in the CGIAR and the National Agricultural Research systems [8]. Further collaborations between CO and Breedbase continuously developed ontologies for several root, tuber, and banana crops (RTB) [9]. In this collaborative effort, both projects provide services for breeding programs to collect, store, and analyze phenotyping data with comprehensive crop-specific ontologies describing a multitude range of measured traits [9].

The core purpose of crop ontologies is to provide terms for measuring phenotypic observations (“trait”) by recording how the trait is measured (“method”) and how the observation is expressed (“scale”). The combination of trait, method, and scale comprises a “variable,” providing breeders with a clear definition of what observation is measured and how [10].

While many plant breeding traits, such as “plant height” or “fruit sugar content,” are fundamentally straightforward in terms of their meaning, method of sampling, and scale or unit of measurement, complexities can arise quickly when more composite sampling strategies are used, for example, when traits are measured at different times or in different plant tissues, subjected to different treatments, or measured repeatedly at different time intervals, breeding cycles, or events. As these sampling strategies can be *ad hoc*, not including such terms in the official pre-composed ontology is often preferred. Different users may want the flexibility to sample the same trait at various time points or contexts and need slightly different sampling parameters [11, 12].

A possible solution to the stability versus flexibility problem is the post-composition approach. In this approach, terms from orthogonal ontologies can be combined to form a new, post-composed term. Orthogonal ontologies are nonoverlapping [13]; in the Breedbase post-composing tool, they describe different domains such as objects, attributes, time points, and breeding events. These terms can be common and shared across databases, such as time terms, or tailored specifically for each crop, such as crop-specific breeding cycles or plant anatomy.

An alternative to post-composition would be to pre-compose all the terms, thus making them part of the official trait ontology. However, pre-composing everything would create an extensive trait ontology that would be unwieldy to handle. In the post-composition approach, only the required and used combinations are generated, keeping the pre-composed ontology smaller, cleaner, and easier to navigate.

Of course, there are other ways to handle the additional information provided with the orthogonal ontologies, for example, by creating observation-specific sample entries to which the additional information can be attached. However, the querying of such extraneous information is not standardized, whereas retrieval of ontology terms and values is a standard query in most databases.

## Materials and methods

### Database interface and schema

The Breedbase code is available as a Docker and can be downloaded from the GitHub repo https://github.com/solgenomics/breedbase_dockerfile. It contains a webserver and the PostgreSQL database comprising most of the modules from the Chado schema [14] and custom schemas tailored for the Breedbase requirements.

**Figure 1.**
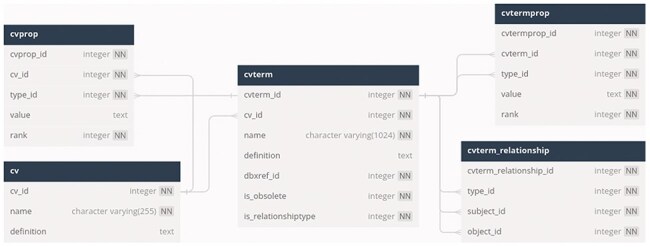
Database schema diagram of the tables required for storing post-composed ontology terms.

**Figure 2.**
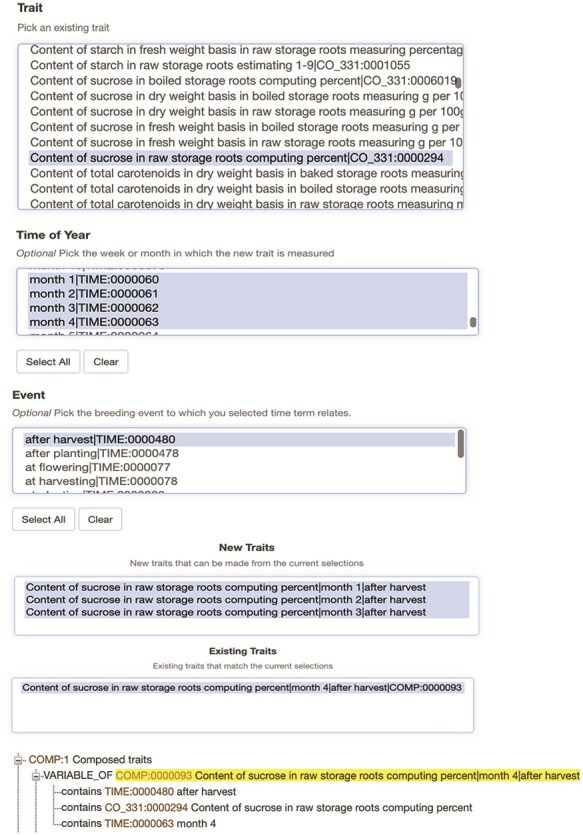
The user interface for post-composing traits from SweetPotatoBase. Selecting the trait ontology term CO_331:0000294 along with time terms “month 1, month 2, month 3, and month 4” and the breeding event term “after harvest.” The interface shows one of the combinations is already stored in the database as a post-composed term, and three may be stored as new traits. The composed trait in the database is displayed with its assigned ID (COMP:0000093) and its three components (variable, time, and event) using a “contained” Relationship Ontology term.

The post-composed terms are stored in the Chado schema cvterm module [15] in a PostgreSQL database. Each trait and orthogonal ontology term is stored in the cvterm table, and the name of the ontology is stored in the cv table. The category of each ontology (trait, time, object, attribute, and event) is stored in the Chado schema “cvprop” table, whereas both the subject–object relationships within each ontology and the combinations of the terms used for building a post-composed trait are stored in the cvterm_relationship table (Fig. 1). The cvterm of each post-composed term is stored as the “object” in cvterm_relationship, and each component of the term is stored as a “subject,” while the object–subject relationship type is the term “contains” from the Relationship Ontology (Fig. 2). The components of the post-composed terms are not ranked, since the post-composing tool allows using only one term from the trait ontology and from each orthogonal ontology when generating a new trait.

### Post-composing data by crop

SQL and R codes used for querying the databases and generating the bar histograms can be found at https://github.com/solgenomics/post-composing-paper.

The first step was to extract the accessions phenotyped with post-composed traits from CassavaBase, YamBase, MusaBase, and SweetPotatoBase using the query below. The post-composed traits have a “COMP” prefix in the database:

-- Use this query to extract the data and then calculate the groups in R
-- Extract accessions phenotyped with post-composed
**SELECT** accessionsxtraits.accession_id **AS id**, accessions.accession_name **AS** accession, **count**(accessionsxtraits.accession_id) **AS** total
**FROM** accessionsxtraits
**INNER JOIN** accessions **ON** accessionsxtraits.accession_id = accessions.accession_id
**WHERE** accessionsxtraits.trait_id **IN** (**SELECT** traits.trait_id **FROM** traits **WHERE** traits.trait_name **LIKE** ‘%COMP%’)
**GROUP BY** accessionsxtraits.accession_id, accessions.accession_name
**ORDER BY** total **DESC**;

Then using R, the different groupings were obtained using the elbow method to find the optimal number of clusters:

# Set working directory
setwd(”∼/Documents/Queries/”)
rm(list = ls())
# Use the file generated from the first query
myDF <- read.csv(”cassava_accessions_
count.csv”, header = **T**, sep = “,”)
head(myDF)
#Elbow Method for finding the optimal number of clusters
set.seed(1234)
# Compute and plot wss for k = 2 to k = 6.
k.max <- 6
data <- as.matrix(scale(myDF$total))
wss <- sapply(1:k.max,
 **function**(k){kmeans(data, k, nstart=50,iter.max = 15 )$tot.withinss})
wss
plot(1:k.max, wss,
	type=“b”, pch = 19, frame = **FALSE**,
 	xlab=“Number of clusters K”,
 	ylab=“Total within-clusters sum of
 squares”)
#From the plot I choose 3 clusters
cl <- kmeans(myDF$total, 3)
cl$centers #Centroid values

After obtaining the different groups, the query below was used to extract traits for each group:

-- This is to get traits for each group
-- With that will be possible to find out which trait root is more frequent
-- After extracting the traits, use R or Excel to check the frequency of the trait root
 **SELECT**
	T.trait_id,
	T.trait_name,
 **COUNT**(AX.accession_id) **AS** appearance_count
 **FROM**
	accessionsxtraits AX
 **INNER JOIN**
	traits T **ON** AX.trait_id = T.trait_id
 **INNER JOIN**
	accessionsxtrials **AT ON** AX.accession_id =
 AT.accession_id
 **WHERE**
	AX.accession_id **IN** (
 **SELECT**
 	AX2.accession_id
 **FROM**
 	accessionsxtraits AX2
 **INNER JOIN**
 	traits T2 **ON** AX2.trait_id = T2.trait_id
 **WHERE**
 	T2.trait_name **LIKE** ‘%COMP%’
 **GROUP BY**
 	AX2.accession_id
 **HAVING** 
**COUNT**(**DISTINCT** AX2.trait_id) >= 23 --
 Set values for each group
	)
**AND** (T.trait_name **LIKE** ‘%COMP%’) -- Filtering out traits containing ‘COMP’
 **GROUP BY**
	T.trait_id, T.trait_name
 **ORDER BY**
	appearance_count **DESC**, T.trait_name;

Then using R, to obtain the most frequent trait roots in the post-composed trait:

## This part is to find out which trait root is more frequent in post-composed trait
## Use the file from the second query
library(stringr)
myDF2 <- **read**.csv(”traits_frequency_ groupA.csv”, header = T, sep = “,”)
myDF2$root_trait <- str_extract(myDF2$trait_name, “[^|]+”)
trait_freq <- **as**.data.frame
(tapply(myDF2$appearance_ count,
myDF2$root_trait, **sum**))
trait_freq <- tibble::rownames_to_column
(trait_freq, “traits”)
colnames(trait_freq)[2] <- “freq”
trait_freq <- trait_freq[**order**(-trait_freq$freq),]
# Printing top 10 **more** frequent root traits
trait_freq[1:10,]

The SQL query below was used to obtain the number of post-composed traits for each group obtained, as was illustrated previously. By changing “LIKE ‘%COMP%’” to “not LIKE ‘%COMP%’”, the total number of pre-composed traits was also obtained for each group:

-- Counting post-composed traits in each group
-- Also is possible to count pre-composed traits in each group
-- getting the total number of traits
 **SELECT** 
 **COUNT**(**DISTINCT** T.trait_id) **AS** total_traits
 **FROM**
	accessionsxtraits AX
 **INNER JOIN**
	traits T **ON** AX.trait_id = T.trait_id
 **WHERE**
	AX.accession_id **IN** (
 **SELECT**
 	AX2.accession_id
 **FROM**
 	accessionsxtraits AX2
 **INNER JOIN**
 	traits T2 **ON** AX2.trait_id = T2.trait_id
 **WHERE**
 	T2.trait_name **LIKE** ‘%COMP%’
 **GROUP BY**
 	AX2.accession_id
 **HAVING**
 	(**COUNT**(**DISTINCT** AX2.trait_id) >= 16 **and COUNT**(**DISTINCT** AX2.trait_id) < 23 ))
 **AND** (T.trait_name **NOT LIKE** ‘%COMP%’);

The total number of accessions for each group was also obtained using the query below:

-- Count accessions in different groups
**SELECT count**(*) **AS** total_lines
**FROM** (**SELECT DISTINCT** accessionsxtraits.accession_id **AS id**,
 accessions.accession_name **AS** accession,
**COUNT**(accessionsxtraits.accession_id) **AS** total
**FROM** accessionsxtraits
**INNER JOIN** accessions **ON** accessionsxtraits.accession_id = accessions.accession_id
**WHERE** accessionsxtraits.trait_id **IN**
(**SELECT** traits.trait_id **FROM** traits
**WHERE** traits.trait_name **LIKE** ‘%COMP%’)
**GROUP BY** accessionsxtraits.accession_id,
accessions.accession_name
**HAVING COUNT**(accessionsxtraits.
accession_id) < 16
) **AS SUBQUERY;**

Finally, the top 10 most used post-composed traits for each crop were obtained. This is a query used for CassavaBase and similar queries were also applied to other databases. These were then used to plot a barplot in R:

**SELECT** o.name **AS object**, t.name **AS type**, s.name **AS** subject, cv.name
**FROM** cvterm_relationship cr
**JOIN** cvterm o **ON** (cr.object_id = o.cvterm_id)
**JOIN** cvterm s **ON** (cr.subject_id = s.cvterm_id)
**JOIN** cvterm t **ON** (cr.type_id = t.cvterm_id)
**JOIN** cv **ON** (s.cv_id = cv.cv_id)
**WHERE** t.name = ‘contains’ **AND** cv.cv_id != (
**SELECT** cvprop.cv_id
**FROM** cvprop
**JOIN** cvterm **ON** (type_id = cvterm_id)
**WHERE name** = ‘trait_ontology’
)**ORDER BY** s.name);

### Post-composing groups (A, B, and C)

The three groups representing the usage of post-composed and pre-composed traits in each instance of Breedbase were calculated following these steps:

1. Counting the number of phenotyping data points for each accession with post-composed traits (data points considered include all experimental levels such as plot, plant, or tissue sample). This resulted in a table with accessions and total data points with post-composed traits.
2. Using an R script, the table with accessions and data points was used to identify clusters with *k*-means.
3. The number of groups identified from *k*-means was three (A, B, and C), and the centroid of each group was used to define the boundaries.

## Results

### The post-composing interface in Breedbase

To create post-composed terms efficiently, we have implemented an easy-to-use user interface. Creating post-composed terms in Breedbase is limited to logged-in users with “submitter” account privileges. In Breedbase, this interface can be accessed by selecting “Compose a New Trait” from the “Analyze” menu. The interface shows several pop-up selectors, one for the pre-composed trait ontology term, and several others for orthogonal ontologies to be combined with the trait ontology, typically including ontologies describing timing, sampling, or sample preparation methodologies (Fig. 2). From each pop-up, terms can be selected, and the combination of these terms, which represents the post-composed term, is shown in a text field below (Fig. 2). If the post-composed term has already been created, it appears in a text area on the lower left; if it is a new, never-before-created term, it appears in a text area on the lower right. The new terms can be selected and stored by clicking a submit button.

The created terms are not validated by database curators, and quality-control is the responsibility of the post-composed trait submitter. This feature allows verified Breedbase users with “submitter” privileges to generate new post-composed traits promptly as required by their breeding program. On the other hand, pre-composed terms can only be added by database curators and require a review by at least a second curator before being added to the official CO.

### User-generated variables in CO and Breedbase

The crop ontologies are a community-driven effort and thus rely on continuous user feedback and curation by designated expert curators. The ontologies used in Breedbase are developed collaboratively with the CO project using specific guidelines for user-generated terms and term requests [10]. The conceptual model defines a pre-composed “variable” as a combination of a trait, method, and scale. Users have several options for submitting new traits and their derived variables:

(1) Using the CO “Trait Dictionary” (TD) template: This option allows a community-nominated curator to submit a curated TD with multiple traits and variables. Curators can define new traits during the growing season and periodically submit a TD to CO for validation. Accepted traits and variables are verified by CO curators, incorporated into the relevant CO, and published at cropontology.org. A repository is also created in the Planteome GitHub (https://github.com/Planteome) [16] to keep track of the TD versioning, provide the TD in both .CSV and .obo formats, and enable submission of terms as an issue.

(2) Submit new traits and variables using a request form trait-requests.planteome.org (or submit.rtbbase.org for root, tubers, and banana crops) to request a small number of traits or to edit an existing trait or variable: Common edits can be changing term definitions and adding or removing synonyms. The form generates a ticket on the Planteome GitHub (https://github.com/Planteome) for the selected crop and assigns a curator to review the request. For requests submitted with a GitHub account, tickets are automatically assigned authorship on their behalf to enable continued communication between curators and requestors.

In Breedbase, post-composed traits can be searched in the “Trait Search” page using text from any component of the term (traits and orthogonal ontologies), and also in the “Ontology Browser” tool using text search, term ID, or by browsing the structure of the post-composed ontology [2].

### CO development of pre-composed traits and variables

Traits and variables are curated before being incorporated into the official ontologies and then reviewed by at least one other curator before being added officially. All edits are stored in the Planteome project GitHub, and it is up to each database, the relevant instance of Breedbase, or CO, to update to the latest version. Variables can then be used for post-composing in Breedbase with additional entities (object, attribute, time, and event), including for cassava [17], banana [18], yam [19], and sweet potato [20]. Importantly, post-composed ontologies should be orthogonal (nonoverlapping). However, ontology curators can decide if it is appropriate to incorporate a post-composed term into the pre-composed ontology, for example, if a term is widely used.

### Usage of post-composed traits in Breedbase

**Figure 3.**
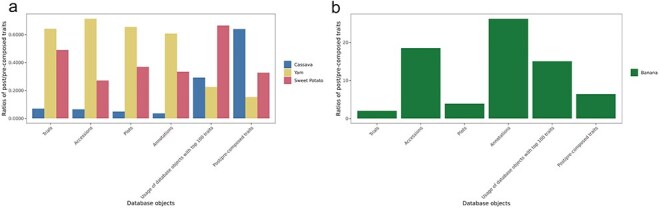
Ratios of post-composed/pre-composed trait annotations by crop. (a) cassava, yam, and sweet potato. (b) Banana. The numeric values for this graph are listed in Table 1

Ontology terms can be used to annotate phenotyping data at several levels. In Breedbase, the phenotyping experimental data are linked to a trial. Within the trial, the observation unit can be a plot, plant, or tissue, and all are linked to a plant accession entry. To show how post-composed traits are used in each instance of Breedbase, the total number of database objects with phenotyping data is summarized in Table 1. The queries for the database objects table were executed in July 2024 and can be found on GitHub (https://github.com/solgenomics/post-composing-paper). The data highlight significant diversity in the number of post-composed traits among different crops, with banana having the highest count at 2303 traits and yam having the lowest at 35. Variability in the number of accessions phenotyped with post-composed traits is evident, ranging from 24 800 for cassava to 7537 for banana (Table 1, Fig. 3).

**Table 1.** Database objects with the number of post-composed/pre-composed traits and their ratio

	Cassava	Banana	Yam	Sweet Potato
Post-/pre-composed traits	(370/579) = 0.639	(2303/359) = 6.415	(35/231) = 0.152	(107/327) = 0.327
Usage of database objects with top 100 traits	(3460/11.8K) = 0.292	(20.1K/1340) = 15.046	(2296 /10.25K) = 0.224	(4506/6772) = 0.665
Trials	(432/6215) = 0.0695	(60/30) = 2	(347/541) = 0.641	(731/1492) = 0.49
Accessions	(24.8K/381K) = 0.065	(7537/407) = 18.518	(11.3K/15.8LK) = 0.713	(32K/118K) = 0.271
Plots	(57.6K/1.18M) = 0.049	(23K/5922) = 3.897	(32.4K/49.6K) = 0.654	(204K/554K) = 0.368
Plants	(270K/25.2M) = 0.107	(4579/0)	(1308/8680) = 0.15	(0/0)
Tissue samples	(1.09M/31.87M) = 0.034	(13.1M/0)	(960/1680) = 0.571	(0 /23K)
Annotations	(1.2M/33.7M) = 0.036	(166K/6359) = 26.192	(46.4K/76.4K) = 0.608)	(236K/697K) = 0.334

### Post-composing by crop

The post-composing tool is tailored to accommodate a collection of crop-specific phenotyping data. Time terms (time of year or day and temporal breeding events) are standard for all crops, while objects and attributes are more crop-specific (Table 2). Currently, terms for “plant section” refer to specific parts of the plant, for example, the distal or proximal part of the root. Terms for “plant level” refer specifically to the rank of the banana “hands” (fruit bunches) and leaves. “Root slice” terms are used for roots or tubers cut into cubes and slices and are currently used by yam breeders. Any post-harvest modifications, such as cooking or mashing, are described by “treatment” terms, and banana has crop-specific attributes for “plant cycle,” describing the plant’s ability to set fruit in multiple consecutive cycles.

**Table 2.** Breedbase orthogonal ontologies for post-composing used for cassava, banana, yam, and sweet potato and the number of post-composed traits using each ontology

	CassavaBase	MusaBase	YamBase	SweetPotatoBase
Plant section (object)	17	–	–	0
Plant level (object)	–	1586	–	–
Root slice (object)	–	–	0	–
Treatment (attribute)	92	–	0	0
Plant cycle (attribute)	–	1901	–	–
Time of day/year	358	1017	32	105
Breeding event	25	2002	0	85

**Figure 4.**
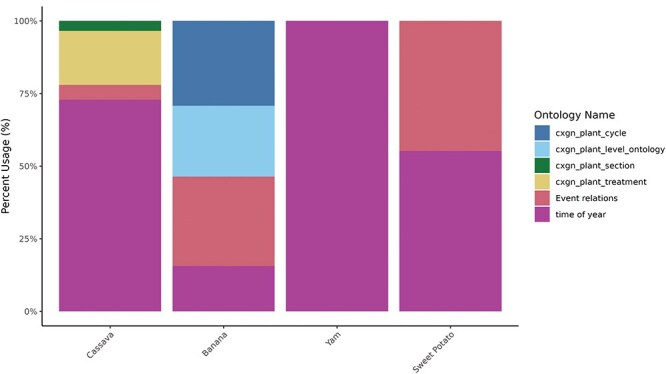
Post-composing traits using orthogonal ontologies by crop. Percentage of usage of each orthogonal ontology (*y*-axis) by crop (*x*-axis). Time of year and event ontologies are common for all crops, while treatment, cycle, plant section, and plant level are crop-specific.

**Figure 5.**
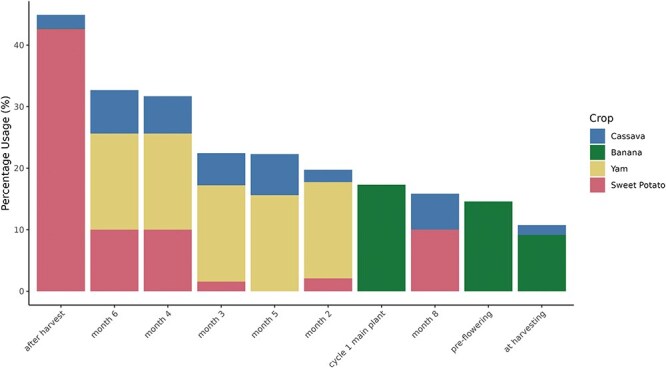
Most used orthogonal ontology terms (*x*-axis) by percentage usage (*y*-axis) in each Breedbase crop.

The current usage of each orthogonal ontology in user-created post-composed terms by crop is shown in Fig. 4, and the 10 most-used terms by crop are shown in Fig. 5. The total usage of each orthogonal ontology in post-composed traits is represented in percentage for normalization purposes since the total number of traits varies significantly between the databases.

The crop trait ontologies are developed and maintained following the CO guidelines and governance framework [10]. They are published on the CO website and are hosted on the Planteome GitHub (https://github.com/Planteome). In contrast, the orthogonal ontologies are Breedbase-specific and stored in the Breedbase GitHub in obo format [21] (https://github.com/solgenomics/sgn/tree/master/ontology). The trait and orthogonal ontologies are loaded into each Breedbase instance using Chado loading scripts (https://github.com/GMOD/chado_tools/blob/master/chado/bin/gmod_load_cvterms.pl). The variations of allowed ontology combinations for post-composing can be set in the backend databases using the “cvprop” table and by setting keys in the Breedbase configuration file.

**Figure 6.**
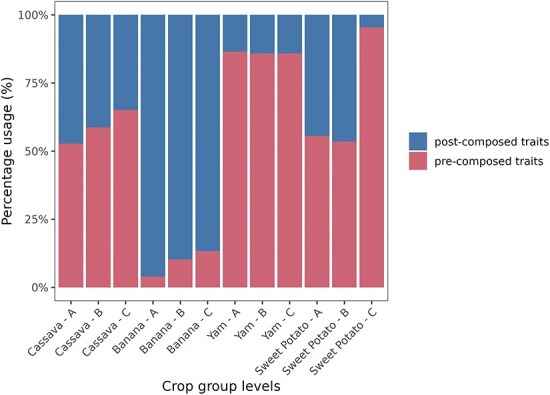
Percentage and total number of unique post-composed and unique pre-composed traits by crop (cassava, banana, yam, and sweet potato) and trait group (A, B, and C).

To represent the usage of post-composed and pre-composed traits in each instance of Breedbase, we categorized the traits used for phenotyping into three distinct groups of traits (A, B, and C) based on the number of unique post-composed traits employed in accession phenotyping. For example, Group A (A ≥ 44) in cassava (Table 3) includes accessions with phenotyping data for 44 or more post-composed traits. These trait groups were identified for each crop through the application of *k*-means clustering (see Materials and methods). In each crop, the number of pre-composed and post-composed traits varies across groups (A, B, and C). Groups with a larger number of evaluated accessions, such as Group C in cassava, which contains 25 602 accessions evaluated for fewer than 22 post-composed traits, are expected to play a more prominent role in the earlier stages of the breeding program. In contrast, Group A, with only 18 accessions, but evaluated for 44 or more traits, is expected to have a more significant impact in the later stages (Fig. 6).

**Table 3.** Comparative analysis of accessions and traits in CassavaBase: post-composed and pre-composed trait usage statistics by grouping clusters A, B, and C

Group	Number of accessions without post-composed traits	Number of accessions with post-composed traits	Number of unique post-composed traits	Number of pre-composed traits
A (≥44)	18 201	18	69	76
B (≥22, <44)	92 794	288	180	260
C (<22)	253 288	25 602	139	278

The total number of accessions phenotyped using each group of traits is represented in the following tables: cassava (Table 3), banana (Table 4), yam (Table 5), and sweet potato (Table 6).

**Table 4.** Comparative analysis of accessions and traits in MusaBase: post-composed and pre-composed trait usage statistics by grouping clusters A, B, and C

Group	Number of accessions without post-composed traits	Number of accessions with post-composed traits	Number of unique post-composed traits	Number of pre-composed traits
A (≥110)	0	59	652	32
B (≥38, <110)	0	1594	280	32
C (<38)	407	5877	209	27

**Table 5.** Comparative analysis of accessions and traits in YamBase: post-composed and pre-composed trait usage statistics by grouping clusters A, B, and C

Group	Number of accessions without post-composed traits	Number of accessions with post-composed traits	Number of unique post-composed traits	Number of pre-composed traits
A (≥23)	11 090	64	25	150
B (≥16, <23)	1215	759	24	145
C (<16)	3248	10 497	26	155

**Table 6.** Comparative analysis of accessions and traits in SweetPotatoBase: post-composed and pre-composed trait usage statistics by grouping clusters A, B, and C

Group	Number of accessions without post-composed traits	Number of accessions with post-composed traits	Number of unique post-composed traits	Number of pre-composed traits
A (≥60)	1496	63	60	76
B (≥48, <60)	16 507	12	60	71
C (<48)	102 442	32 695	6	131

### Post-composing traits in conjunction with analysis tools in Breedbase

Post-composing of terms is also used internally by Breedbase to help track how data have been transformed by analysis methods. Raw data are usually associated with variables that can be post-composed or not. However, when an analysis is run, such as a mixed model, on a variable such as plant height, the resulting data point is not a raw data value anymore which should be reflected in the ontology. For this purpose, Breedbase contains an ontology describing analyses, called the SGN Statistics Ontology (SGNSTAT), with a term for each possible analysis in the system. For example, when a mixed model analysis is run with Linear Mixed-Effects Regression (LMER) that yields adjusted means from Best linear unbiased prediction (BLUPs) and stored in the database, the terms associated with the data are post-composed with the SGNSTAT term SGNSTAT:0000034 (adjusted means from BLUPs using LMER). Multiple steps can be in theory chained together, as required by the analysis pipeline run. Although this system provides clarity about how a data point has been processed, it has the downside of making the terms unwieldy long.

## Discussion

The use of databases has become a fundamental part of crop improvement as more breeders look for computational tools for collecting, storing, and analyzing phenotypic and genotypic data. Using trait ontologies for phenotyping is a crucial part of data standardization, as the same terminology can be used across breeding programs, enabling data analysis across trials and databases. While the existing crop ontologies are the backbone for phenotyping standardization, they are not optimized for capturing more granular phenotypes or a large number of repeat measurements by time intervals, plant parts, breeding cycles, treatments, and combinations of such attributes. To address these issues, Breedbase allows users to generate post-composed terms on the fly directly from the web interface using a number of predefined orthogonal ontologies.

We presented data for four instances of Breedbase, which deal with root and tuber crops (cassava, banana, yam, and sweet potato), and for which users have generated and utilized post-composed traits. These traits had been used for phenotyping along with the predefined trait ontologies that exist for each crop.

While the flexibility of creating post-composed traits instantly from the front end is an advantage for a dynamic breeding program, issues can arise with not having a curation step by a professional curator. Breedbase offers tutorials, manuals, email support, and workshops for user support and training on how to use the system, with an effort to train users on how to use the post-composition tool, reuse existing traits when possible, and submit new traits when there is phenotyping data to associate. Post-composing is not a substitute for curating the base ontologies, as new concepts cannot be captured with post-composing alone, but need to be reflected in a base ontology. However, expansion of terms in the orthogonal ontologies, which are expanded only when there is a request from a user, can take time and has to go through the official process. The post-composed terms are stored only locally in each Breedbase, which limits the ability to reuse those traits in other databases. Unlike pre-composed trait ontologies, post-composed terms are confined to be used only in the database in which they are generated.

The work described in this paper demonstrates use cases for trait post-composing in four root or tuber crops: yam, banana, cassava, and sweet potato. The framework for post-composing includes the crop trait ontology term combined with predefined internal orthogonal ontologies describing temporal terms, treatments, breeding cycles, and plant anatomy. The orthogonal ontologies are highly customizable and terms can be added to a Breedbase instance as needed by users.

The trait usage data we have shown (Tables 1 and 3–6) demonstrate clearly that the post-composing method is useful and heavily used in the databases analyzed. There are significant differences between the databases; the database with the most post-composed terms is MusaBase (banana), because almost every term is post-composed with the concept of “cycle” in which each trait combination is multiplied by the plant’s fruiting cycle number. Banana breeders also use the concept of “hand rank” to indicate the position of the fruit bunch on the tree.

CassavaBase has the largest number of annotated observation units, using both pre-composed and post-composed traits, since it is the database with the largest number of users, and the data submitted dates back the longest.

In the more advanced stages of breeding programs, a smaller number of accessions are expected to be evaluated. On the other hand, the complexity in the number of traits increases to ensure that the released cultivar is validated for the expected characteristics [22]. In this context, the use of post-composed traits is differentiated into three groups (A, B, and C). Group A has fewer evaluated accessions but a higher number of post-composed traits. Notably, in MusaBase, all accessions in Groups A and B were evaluated with post-composed traits. This demonstrates that the focus and decision of the banana breeding program is on post-composed traits.

In contrast, the cassava program has 17 434 accessions without post-composed traits in Group A and only 18 accessions with post-composed traits, indicating that the focus of the cassava program is on pre-composed traits. Additionally, the number of accessions increases in Groups B and C, suggesting that post-composed traits in cassava are more widely distributed across the different stages of the breeding program.

The yam (Table 5) and sweet potato (Table 6) programs have a smaller number of post-composed traits, ranging from 20 to 60 post-composed traits in each group (A, B, and C), compared to the cassava and banana programs, which have numbers ranging from 69 to 264. This smaller number may indicate that yam and sweet potato are still in the early stages of using post-composed traits.

Grouping accessions based on the number of post-composed traits can provide valuable insights for directing future investments in breeding programs. By understanding how many accessions exhibit a higher number of desirable traits, breeders can identify trends and gaps in the breeding process. This information allows for a more strategic allocation of resources, ensuring that breeding efforts are concentrated on stages or groups with the greatest potential for improvement.

Post-composition of terms is a valuable tool for balancing standardization and providing flexibility of ontologies serving a wide variety of users with various needs. Our work of managing multiple instances of Breedbase, as a digital ecosystem for plant breeders, demonstrates the need for robust, manually curated crop ontologies while allowing users to post-compose ontology terms tailored for specific and granular use cases.

This system allows building a crop-specific, highly customizable, and user-driven tool. Adding new options and terms in each orthogonal ontology can be done promptly. Additional levels of granularity can also be added and tailored to specific experimental designs. When the end-user generates new post-composed terms, these are instantly stored in the database, are available for use, and can be shared with other users of the crop-specific database.

Breedbase is a scalable system that is constantly maintained and adopted by new users and crops. The post-composing tool can also be expanded for the needs of different breeding programs by adding more available trait combinations and new orthogonal ontologies for different levels of phenotyping.

## Data Availability

The Breedbase code is open source and available at github.com/solgenomics/breedbase_dockerfile. The crop ontologies used in Breedbase are available at GitHub github.com/Planteome.
